# JNK pathway inhibition selectively primes pancreatic cancer stem cells to TRAIL-induced apoptosis without affecting the physiology of normal tissue resident stem cells

**DOI:** 10.18632/oncotarget.7066

**Published:** 2016-01-28

**Authors:** Alejandro Recio-Boiles, Matthias Ilmer, P. Robyn Rhea, Claudia Kettlun, Mitja L. Heinemann, Jennifer Ruetering, Jody Vykoukal, Eckhard Alt

**Affiliations:** ^1^ Department of Translational Molecular Pathology, The University of Texas MD Anderson Cancer Center, Houston, Texas, USA; ^2^ Department of General Oncology, The University of Texas MD Anderson Cancer Center, Houston, Texas, USA; ^3^ InGeneron Incorporated, Houston, Texas, USA; ^4^ Department of Medicine, Tulane University Health Science Center, New Orleans, Louisiana, USA; ^5^ ISAR Klinikum, Munich, Germany

**Keywords:** pancreatic cancer, cancer stem cells, JNK, TRAIL, apoptosis

## Abstract

**Objective:**

Successful treatment of solid cancers mandates targeting cancer stem cells (CSC) without impact on the physiology of normal tissue resident stem cells. C-Jun N-terminal kinase (JNK) signaling has been shown to be of importance in cancer. We test whether JNK inhibition would sensitize pancreatic CSCs to induction of apoptosis via low-dose TNFα-related apoptosis-inducing ligand (TRAIL).

**Design:**

Effects of JNK inhibition (JNKi) were evaluated *in vitro* in functional assays, through mRNA and protein expression analysis, and in *in vivo* mouse studies. CSCs were enriched in anoikis-resistant spheroid culture and analyzed accordingly.

**Results:**

We confirmed that the JNK pathway is an important regulatory pathway in pancreatic cancer stem cells and further found that JNK inhibition downregulates the decoy receptor DcR1 through IL-8 signaling while upregulating pro-apoptotic death receptors DR4/5, thereby sensitizing cells - even with acquired TRAIL-resistance - to apoptosis induction. Treatment of orthotopic pancreatic cancer xenografts with either gemcitabine, JNKi or TRAIL alone for 4 weeks showed only modest effects compared to control, while the combination of JNKi and TRAIL resulted in significantly lower tumor burden (69%; *p* < 0.04), reduced numbers of circulating tumor cells, and less distant metastatic events, without affecting the general health of the animals.

**Conclusions:**

The combination of JNKi and TRAIL significantly impacts on CSCs, but leaves regular tissue-resident stem cells unaffected – even under hypoxic stress conditions. This concept of selective treatment of pancreatic CSCs warrants further evaluation.

## INTRODUCTION

Pancreatic ductal adenocarcinoma (PDAC) is typically associated with drug resistance, metastasis, and dismal clinical outcomes. Currently, surgery is the only potentially curative therapeutic option for PDAC patients [1]. Early detection of this stroma-rich, desmoplastic neoplasm is challenging because of long symptom-free intervals [2]. Although extensive efforts have been made to advance the molecular and clinical understanding of PDAC, drug-based treatment regimens have been unsatisfactory so far, and 5-year survival has improved only slightly over the past decades.

PDAC is associated with several well-described mutations in a subset of genes including those that encode *KRAS*, *SMAD4*, and *p53* [3] and exhibits additional mutations that affect various pathways [4]. Spontaneous genetic alterations make successful treatment relatively difficult since they provide pancreatic tumors with means to escape from available therapies. The c-Jun N-terminal kinase (JNK) pathway is one of the pathways activated in PDAC. Its transcription factor c-Jun can be induced by cellular stress, e.g., hypoxia or inflammatory signals, and regulates, among other cellular processes, apoptosis [5]. JNK1, through inhibition of apoptosis, and JNK2, via activation of AKT, increase tumor cell survival. Both isoforms are implicated in endothelial attachment, and promotion of barrier disruption by JNK3 can result in extravasation of circulating tumor cells (CTCs). The various JNK isoforms also play roles in metastatic niche remodeling and colonization. In light of such multiplicity, pan-isoform JNK inhibition might prove especially efficacious in the context of cancer therapy [6]. Moreover, it has previously been shown that JNK is frequently active in PDAC downstream of oncogenic KRAS [7] and that inactivating the JNK signaling via different mechanisms can increase apoptosis induction in some hepatocellular carcinoma cells. JNK signaling also plays a critical role in regulating self-renewal and tumorigenesis in cancer stem cells (CSCs) in glioma [8] and has recently been shown to maintain pancreatic CSCs downstream of mutated KRAS [9].

Many types of solid tumors have been found to be heterogeneous and to have a hierarchical organization that is driven by CSCs. CSCs exhibit remarkable abilities for self-renewal, tumorigenesis, drug resistance, and adaptability to changing microenvironments. As such, CSCs are considered the drivers of drug resistance and metastasis [10-12].

The current study was designed to identify selective molecular pathways that would be highly effective in inhibiting cancer growth, specifically that of cancer stem cells. We questioned if JNK signaling plays a pivotal role in differentiated PDAC and, in particular if it would play a role to an even greater extent in pancreatic CSCs. Previously, inhibition of JNK alone has proven to be of limited value in inhibiting cancer cell growth.

In this study we aimed to identify a possible pathway critical for downregulation of the decoy TRAIL receptors 1 and 2 (DcR1/2) without affecting the physiology of normal tissue-resident stem cells even under hypoxic conditions that resemble the desmoplastic environment of PDACs [13]. Accordingly, we evaluated the concept of low-dose JNK inhibition combined with low-dose TRAIL as a possible novel and selective therapeutic approach for pancreatic cancer stem cells.

## RESULTS

### PDAC depends on JNK signaling for growth and survival

JNK is a stress-responsive kinase that is involved in apoptosis, tumorigenesis, and other signaling events [6]. To understand the role and mechanism of JNK in PDAC, we treated (five) different well-characterized pancreatic cancer cell lines with JNK inhibitors SP600125 and JNK-IN-8 at concentrations between 0.5 and 20 μM, thus spanning a range more than 20-fold lower than that typically employed for *in vitro* studies with these compounds [14, 15, 16]. Recent discoveries describe JNK-INH-8 as the first extremely potent and irreversible JNK inhibitor that forms a covalent bond with a conserved cysteine. Moreover, its superior selectivity compared to prior inhibitors suggests that this compound will be useful for future pharmacological approaches of JNK-dependent cellular phenomena requiring further testing [16]. SP600125 was shown in previous publications to be a selective inhibitor of JNK, exhibiting 300-fold selectivity for JNK compared to related MAP kinases ERK2 and p38-2 and the unrelated serine threonine kinase PKA [17-20].

**Figure 1:**
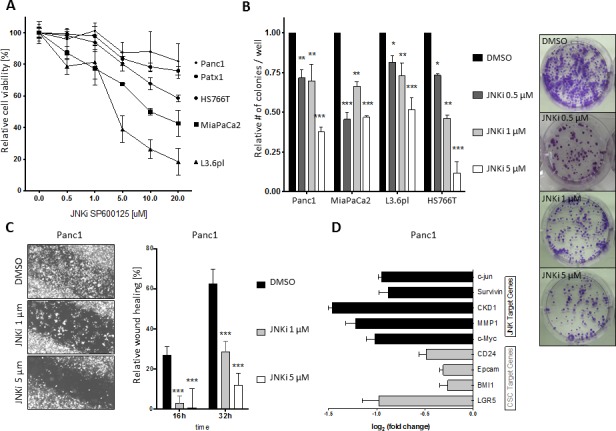
PDAC depends on JNK signaling for growth and survival **A.** Cell viability of Panc1, Patx1, HS766T, MiaPaCa2, and L3.6pl pancreatic cancer cells was determined by MTT assay after treatment with increasing doses of JNKi SP600125 for 24 hours. **B.** Colony formation was evaluated after 10-day incubation of cells with the indicated JNKi concentrations in 6-well plates and staining of colonies with crystal violet. A representative brightfield picture with MiaPaCa2 cells is shown on the right (these pictures are part of Figure S1A). **C.** Bright-field micrographs of scratch migration assay in Panc1 cells after 16 hours (original magnification 5x; left panel). Four different fields were analyzed per time point and treatment. Shown are the mean lengths of the gap closure in relation to the length of the original closure determined at 0 hours. **D.** Expression of JNK target genes (black bars) and CSC target genes (grey bars) in JNKi-treated cells (0.5 μM) relative to expression in untreated cells. Values of genes were standardized to the respective values of housekeeping genes. Experiments were performed in triplicate. *p < 0.05. **p < 0.01. ***p < 0.001.

Low-dose treatment with SP600125 or JNK-IN-8 (0.5 μM or 1.0 μM) resulted in nonsignificant, relatively negligible effects on cell viability in Panc1, MiaPaca2, L3.6pl, Patx1, and HS766T cells (Figure 1A and Supplementary Figure S1A). High-dose treatment (5.0 μM, 10.0 μM, or 20.0 μM) was followed by markedly decreased cell viability in all five cell lines after 24 hours.

Next, we determined the effects of JNKi on clonogenic growth behavior using colony-forming assays. Quantitative analysis after 10 days from last intervention revealed a dose-dependent inhibition of both the number of colonies formed in all cell lines (Figure 1B, left side) and a reduction in colony size, noteworthy to be significant at low-dose JNKi (0.5 μM) not seen on cell viability (Figure 1B, right side, and Supplementary Figure S1B).

Because JNKs are involved in stress-induced processes, we performed scratch closure assays on 2-D monolayers of pancreatic cancer cells [21, 22]. Close to the scratch margins, activated phospho-c-Jun, a downstream member of the JNK pathway, was shown to be activated after 24h (Supplementary Figure S1C, upper row). JNKi inhibited closure in a dose-dependent manner (Figure 1C, left panel), as well as phosphorylation of c-Jun (Supplementary Figure S1C, lower row). It is noteworthy that even at low-dose (1 μM), JNKi had significant effects in scratch closure models for up to 32 hours (Figure 1C, right panel and Supplementary Figure S1C, right panel). Furthermore, low-dose JNKi (0.5 μM) substantially inhibited invasive behavior of Panc1 and L3.6pl cells in a three-dimensional matrigel-coated Boyden invasion chamber assay (Supplementary Figure S1D).

Finally, to shed light on the mechanistic background, we performed qRT-PCR for established JNK target genes, including *c-Jun*, *Survivin, CKD1, MMP1*, and *c-Myc*, in untreated and low-dose (0.5 μM) JNKi-treated pancreatic cancer cells [23]. As expected, JNK target genes (*cJun, Survivin, CKD1, MMP1, c-Myc)* were significantly downregulated after JNKi treatment (Figure 1D and Supplementary Figure S2A). CSC target genes (*CD24*, *EpCAM, BMI1,* and *LGR5*) were also downregulated after JNKi treatment (Figure 1D and Supplementary Figure S2A), suggesting that the JNK pathway might play a role in the regulation of CSCs in pancreatic cancer.

### JNK inhibition attenuates stemness potential of PDAC

**Figure 2:**
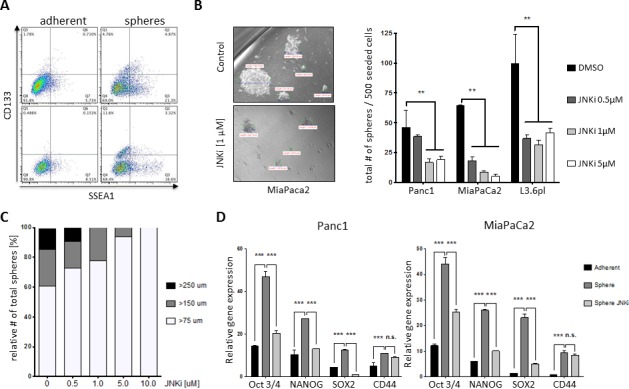
JNK inhibition attenuates stemness potential of PDAC **A.** Flow cytometric analysis of CSC markers CD133 and SSEA1 on adherent pancreatic cancer cells (left) and spheres (right) in L3.6pl (upper panel) and Panc1 (lower panel). **B.** Brightfield micrographs (original magnification x20) of control and JNKi-treated pancreatic spheres. The bars show the actual size of each sphere. Sphere-forming ability after treatment with JNKi at different doses is shown in the bar graphs to the right. Spheres were counted after 10 to 12 days. Experiments were performed in triplicate (right panel). **C.** Relative size distribution of spheres in **B.**. **D.** qRT-PCR of CSC markers in parental Panc1 and MiaPaCa2 cells, spheres, and spheres treated with JNKi (0.5 μM). Experiments were performed in triplicate. **p < 0.01. ***p <0.001.

Since JNKi seemed to inhibit known CSC target genes (Figure 1D and Supplementary Figure S2A), we investigated the role of JNK in pancreatic cancer stemness in more detail. One accepted model for enriching cells exhibiting CSC characteristics is tumorsphere culture [11, 24]. In line with previous reports, we found that pancreatic cancer sphere cells were highly enriched in stem cell markers such as CD133 and SSEA1 (Figure 2A) and preferentially showed higher Wnt activity [25].

Sphere-forming ability is often used as a quantitative estimate of the number of CSCs within a tumor cell population. Similar to JNKi-induced reduction in colony formation (Figure 1B and Supplementary Figure S1B), recurring JNKi administration at concentration as low as 0.5 μM significantly reduced the number of spheres in MiaPaca2 and L3.6pl, while Panc1 required 1 μM (Figure 2B). Strikingly, evaluation of sphere-forming ability in viable cells following single-dose JNKi revealed drastically reduced sphere number, suggesting an enduring effect on functional cancer stemness properties by JNKi (Supplementary Figure S2B). Moreover, in all cell lines analyzed, JNKi also reduced sphere size considerably (Figure 2C). Of note, JNKi exhibited a dose-dependent impact on relative sphere sizes: 40% of spheres in the control group were larger than 150 μm in diameter, compared to 0% in the high-dose treatment group (Figure 2C).

To further understand the effects of JNKi on cancer stemness, we carried out qRT-PCR for CSC markers *Oct3/4*, *Nanog*, *Sox2*, and *CD44*. As expected, we found that expression of these CSC markers was significantly higher in spheres compared to parental cancer cells (Figure 2D and Supplementary Figure S2C). However, low-dose (0.5 μM) JNKi decreased the expression of these CSC markers to levels closer to those detected in parental cells, suggesting that JNKi-treated cells partially lose their CSC phenotype in sphere culture.

### JNKi sensitizes PDAC cells and CSCs to the pro-apoptotic effects of TRAIL

**Figure 3:**
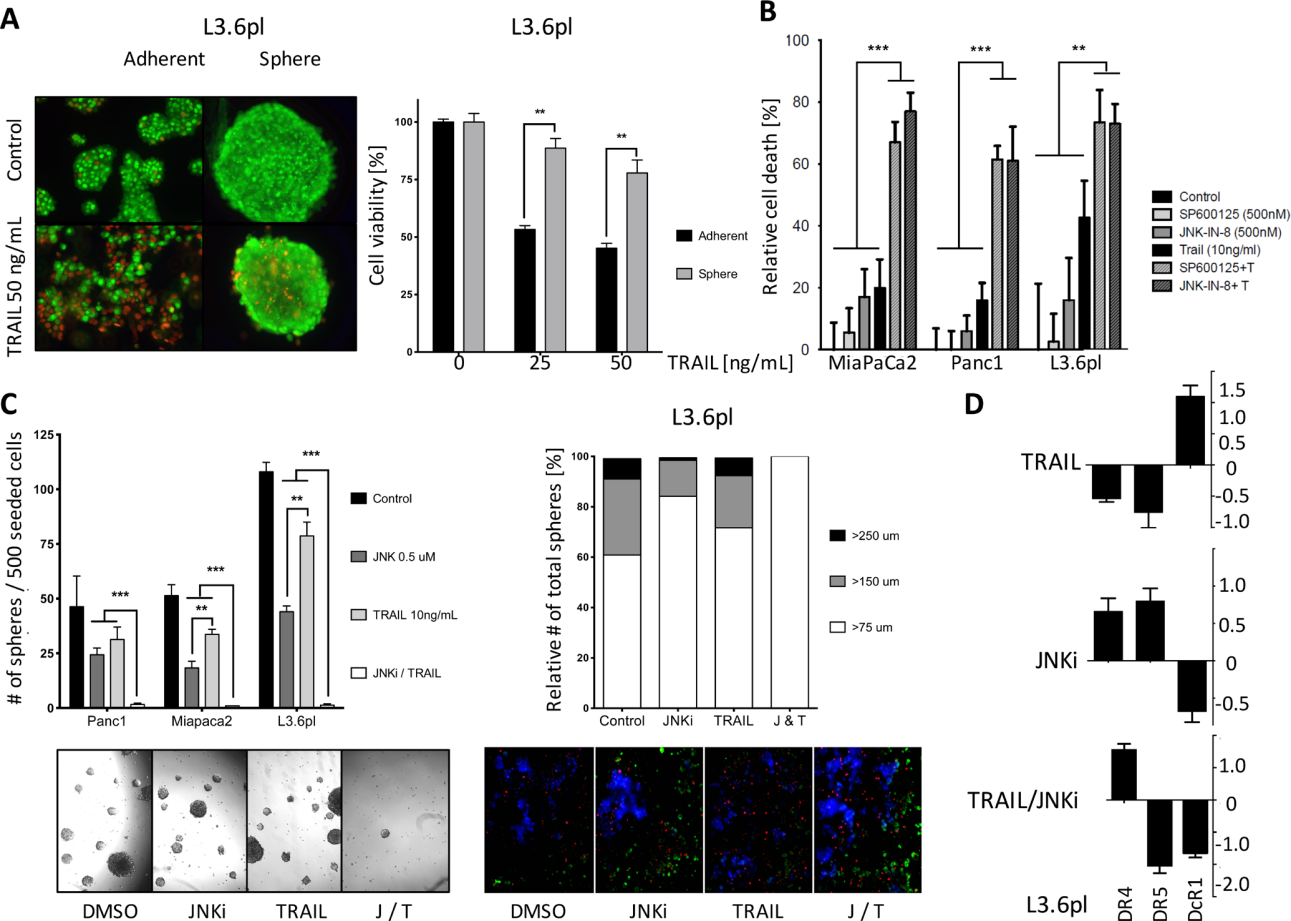
JNKi sensitizes PDAC cells and CSCs to the pro-apoptotic effects of TRAIL **A.** Acridine orange/ethidium bromide staining of adherent and sphere cells after 24 hours of TRAIL treatment (50 ng/mL). Green, live cells; red, dead cells (pictures on the left). MTT assays of adherent and sphere cells after treatment with TRAIL for 24 hours. (bar graph on the right). **B.** MTT assays of parental Panc1, MiaPaCa2, and L3.6pl cells treated with JNKi (SP600125 and JNK-IN-8), TRAIL, or both for 24 hours. Experiments were performed in triplicate. **C.** Total number of spheres (left panel) and relative size distribution of spheres (right panel). Brightfield micrographs of sphere-forming ability (lower left panel) and fluorescence microscopy of apoptosis activation (green) with JNKi, TRAIL, or both (J/T) after 10 days and 1 day respectively in L3.6pl (10x magnification, lower right panel). **D.** Gene expression of *DR4, DR5,* and *DcR1* after treatment with TRAIL, JNKi or both for 24 hours. Shown are the relative values compared to untreated controls. *β-Actin* served as a housekeeping gene. Experiments were performed in triplicate. **p < 0.01. ***p <0.001.

TRAIL is a subject of excitement in the field of cancer therapy. Cancer cells exhibit increased expression of the TRAIL receptors (death receptors DR4 and DR5) compared to non-malignant cells; thus, TRAIL is a natural apoptosis inducer with a preferential effect on cancer cells [26]. We first investigated whether CSC-enriched pancreatic cancer spheres are susceptible to TRAIL. To do so, we used acridine orange/ethidium bromide staining. In L3.6pl cells, parental cells treated with 50 ng/mL TRAIL demonstrated robust apoptosis at 24 hours (Figure 3A, lower left), whereas spheres treated with the same regimen were almost entirely viable (Figure 3A, lower right). MTT viability assay showed that spheres were significantly more resistant to TRAIL-induced cell death than were parental cells at both TRAIL concentrations tested: 25 ng/mL and 50 ng/mL (Figure 3A, right side), highlighting the important role of cancer stem cells as drivers of tumor growth and resistance to treatment.

MTT cell viability assays showed that low-dose JNKi (0.5 μM) alone and low-dose TRAIL (10 ng/mL) alone exerted only a modest effect on cell viability in parental pancreatic cancer cells (Figure 3B).

Since we had observed a substantial reduction of stemness potential following treatment with JNKi (Figure 2), we studied the effects of combining JNKi treatment with TRAIL treatment. We assumed that a combination of low-dose JNKi (0.5 μM) and TRAIL (10 ng/mL) would be clinically achievable, beneficial and tolerable with no or little side effects. The combination of JNKi (with either SP600125 or JNK-IN-8) and TRAIL induced a robust reduction in cell viability in all three cancer cell lines tested: Panc1, MiaPaCa2, and L3.6pl (Figure 3B).

We extended our experiment to CSC-enriched spheres and found that JNKi alone reduced sphere size to some extent, as expected, but that the low-dose combination of JNKi (0.5 μM) and TRAIL (10 ng/mL) completely inhibited sphere growth (Figure 3C, left panel). Quantification of the total sphere number showed that the low-dose combination of JNKi and TRAIL significantly reduced the total number of spheres to a minimum (Figure 3C, left panel). Relative quantification of the sphere sizes showed that treatment with the combination of JNKi and TRAIL only allowed growth of the smallest spheres (>75 μm) (Figure 3C, right panel) suggesting a substantial inhibition of CSC proliferation. Additionally, JNKi and TRAIL showed an increased apoptosis activity compared to TRAIL as indicated by fluorescence microscopy visualization of FITC Annexin V apoptosis detection assays (Figure 3C, right lower panel).

Mechanistically, we found by qRT-PCR that TRAIL treatment (10 ng/mL) alone reduced the presence of the apoptosis receptors DR4 and DR5 compared to control samples (Figure 3D, left upper panel) and increased the expression of the decoy receptor DcR1. This suggests that TRAIL resistance of CSCs is based on increased expression of the decoy receptor DcR1, thus effecting increased survival after TRAIL treatment as binding of TRAIL to DcR1 averts induction of apoptosis. In contrast, treatment with JNKi (0.5 μM) was shown to reverse the phenomenon by increasing the expression of TRAIL death receptors DR4 and DR5 and reducing expression of the TRAIL decoy receptor DcR1 (Figure 3D, right upper panel, and Supplementary Figure S2D). Thus, combination treatment with JNKi enables TRAIL to induce apoptosis in the CSCs, and explains the sensitization of CSCs by JNKi to TRAIL therapy. Regulation of the gene expression of DcR1 and DR4/5 in L3.6pl incubated with both TRAIL and JNKi for 24 hours strengthens the concept that JNK inhibition counteracts increased expression of DcR1 and decreased DR4 (not seen at DR5) level following TRAIL treatment thereby enabling TRAIL to induce apoptosis in the adherent and pancreatic cancer stem cells (Figure 3D, lower panel).

### Even PDAC cells with acquired TRAIL resistance can be re-sensitized by JNK treatment to TRAIL-induced apoptosis

**Figure 4:**
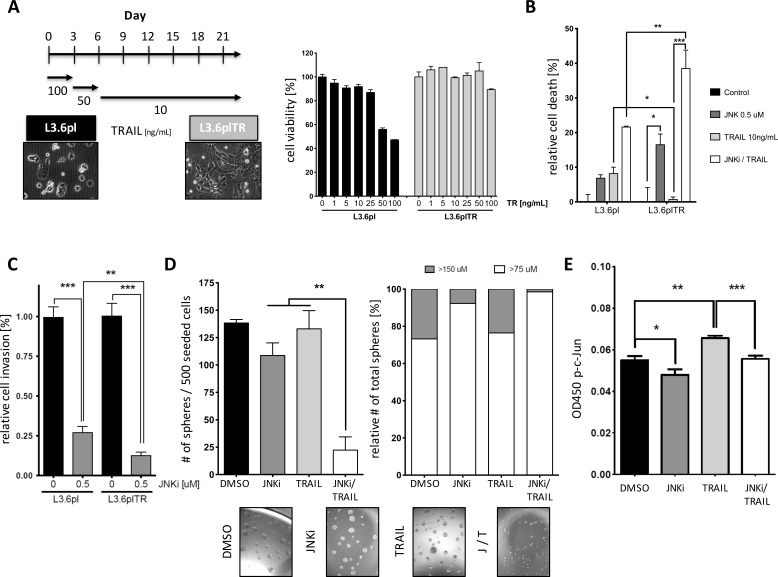
PDAC cells with acquired TRAIL resistance can be re-sensitized by JNK treatment to TRAIL-induced apoptosis **A.** Schematic of the conversion of TRAIL-susceptible L3.6pl cells to TRAIL-resistant L3.6plTR cells. L3.6pl cells were initially treated with high-dose TRAIL to induce rapid cell death of susceptible cells and then maintained in low-dose TRAIL for up to 21 days (left). MTT assay on L3.6pl cells and L3.6plTR cells after treatment with increasing doses of TRAIL (right). **B.** MTT assay on L3.6pl cells and L3.6plTR cells after 24-hour treatment with JNKi, TRAIL, or both (JNKi/TRAIL). **C.** Relative invasion of L3.6pl and L3.6pl TR cells. Invading cells were counted in four different view fields and presented as the mean ± SD. **D.** Brightfield micrographs of sphere-forming ability of L3.6plTR cells treated with JNKi, TRAIL, or JNKi/TRAIL for 10 days (5x magnification) (lower panel). Quantitative analysis of total sphere number (left panel) and sphere size distribution (right panel). **D.** Sandwich ELISA of p-JNK in L3.6plTR cells after 24-hour treatment with JNKi, TRAIL, or JNKi/TRAIL. Experiments were performed in triplicate. *p < 0.05. **p < 0.01. ***p < 0.001.

To mimic the TRAIL-resistant behavior of CSCs, we artificially created a TRAIL-resistant cell line from parental L3.6pl cells, which inherently are highly sensitive to TRAIL. The regimen for inducing TRAIL resistance is described schematically in Figure 4A. Morphologically, cells changed from an epithelial phenotype (Figure 4A, left) to a mesenchymal phenotype (Figure 4A, right) similar to that observed in Panc1, an intrinsically TRAIL-resistant cell line. Compared to parental cells, TRAIL-resistant L3.6pl cells (L3.6plTR) were more resistant to TRAIL and showed detectable cell death only in small numbers of cells, even at TRAIL doses up to 100 ng/mL (Figure 4A, right panel).

Next, we investigated the effect of our established regimen of low-dose JNKi (0.5 μM) and TRAIL (10ng/ml) on L3.6plTR cells. JNKi significantly reduced cell viability in L3.6plTR cells, and to our surprise, the combination of JNKi with TRAIL induced cell death in up to 40% of cells, a significantly greater percentage than observed with the combination treatment in the parental L3.6pl cell line (Figure 4B). Additionally, low-dose JNKi (0.5 μM) significantly inhibited invasive behavior of L3.6plTR cells (87%) when compared to the respective parental cells and significantly more to TRAIL-sensitive L3.6pl (73%) (Figure 4C). As with TRAIL-sensitive L3.6pl spheres (Figure 3C), we observed that CSC-enriched L3.6plTR spheres were highly susceptible to the combination of JNKi (0.5 μM) and TRAIL (10 ng/mL) with respect to both total sphere number (Figure 4D, left panel) and sphere size (Figure 4D, right panel and pictures). With JNKi treatment and even more with the combination treatment, almost all of the spheres formed were smaller than 75 μm in diameter (Figure 4D, right panel).

Sandwich ELISA of p-JNK revealed that JNKi treatment reduced p-JNK expression and TRAIL treatment resulted in increased p-JNK levels in L3.6plTR cells compared to untreated control cells (Figure 4E). The combination of JNKi and TRAIL blocked this increase (Figure 4E), presumably impeding an important apoptosis escape mechanism of TRAIL-resistant cells.

### JNKi does not affect physiology and function of normal tissue-resident stem cells

Currently available cancer treatment regimens often have a limited effect, especially on cancer stem cells, but nevertheless affect the physiology of normal, rapidly growing and dividing cells in the intestinal epithelium or on regular adult tissue-resident stem cells to a degree that induces unwanted side effects or precludes higher dosing. To pre-clinically test whether our approach would be later associated with possible clinically relevant side effects, we isolated human adipose tissue-derived stem cells (ASCs) as reported before [27] in a first step and subjected them to increasing doses of JNKi. Proliferation was only affected at unphysiologically high doses of 10.0 μM or 20.0 μM, and, even then, cell proliferative capacity was only 20% lower compared to untreated ASCs (Supplementary Figure S3A).

To understand whether and how the combination of JNKi and TRAIL would affect cell survival, we treated ASCs with doses of JNKi and TRAIL up to five times of those used in low-dose pancreatic cancer treatment regimens. We found no differences in cell survival compared to control cells (Supplementary Figure S3B). Most importantly, ASCs were functionally unimpaired by JNKi, TRAIL, or their combination as determined by differentiation assays along the mesodermal lineage into osteoblasts (Supplementary Figure S3C, upper panel, Alizarin Red), chondrocytes (middle panel, Alcian Blue), or adipocytes (lower panel, Oil Red O). These data suggest that adult stem cells are unaffected in their cell physiology and multipotent differentiation potential by individual or combination treatment with JNKi and TRAIL.

Because the microenvironment of pancreatic cancers is very desmoplastic, tumors tend to be hypoxic [28]. To simulate these conditions, we cultured the pancreatic cancer cells L3.6pl and L3.6plTR as well as hASCs under hypoxic conditions and then evaluated their response to DMSO, JNKi, TRAIL, or a combination thereof after 48h of exposure. Of note, hASCs were completely unaffected, whereas 60% of L3.6pl and 40% of L3.6plTR could be detected to be in early or late apoptosis by Annexin V-FITC/PI staining (Supplementary Figure S3D). The selectively increased induction of apoptosis in cancer stem cells and the absence of effects on regular normal tissue resident stem cells is a substantial finding with regard to avoiding conceivable side effects for a potential clinical application.

### TRAIL resistance is mediated by autocrine IL-8 downstream of JNK

**Figure 5:**
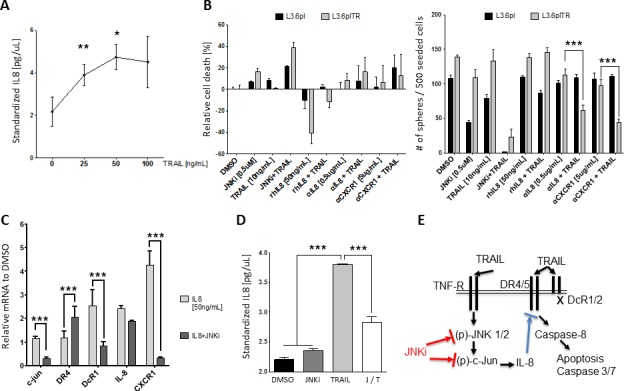
TRAIL resistance is mediated by autocrine IL-8 downstream of JNK **A.** ELISA of IL-8 after treatment of Panc1 cells with TRAIL for 24 hours. **B.** MTT cell viability assay in L3.6pl cells (black) or L3.6plTR cells (grey) after treatment with different combinations of JNKi, TRAIL, IL-8 (rhIL-8), αIL-8, and αCXCR1 (left). Sphere-forming ability with the same conditions was counted after 10 days (right). **C.** Gene expression after treatment with IL-8 or JNKi and IL-8. Shown are the relative values compared to values in untreated pancreatic cancer cells. Experiment was performed in triplicate. **D.** ELISA of IL-8 after treatment with JNKi, TRAIL, or both (left). Experiments were performed in triplicate. **E.** Graphical illustration of how TRAIL, JNK, and IL-8 signaling work together in our model (right).*p < 0.05. **p < 0.01. ***p <0.001.

After establishing the critical role of JNK for pancreatic cancer stem cells, we tried to better understand the underlying molecular connection between JNK activation and TRAIL resistance. IL-8 was reported to attenuate TRAIL sensitivity by upregulating the endogenous Caspase-8 inhibitor cFLIP in prostate cancer cells [29]. We treated pancreatic cancer cells with increasing doses of TRAIL for 24 hours and determined the IL-8 secretion by ELISA (Figure 5A). The IL-8 production increased dose-dependently and exhibited a peak secretion at sublethal TRAIL levels (50 ng/mL in Panc1 cells). Next, we explored whether addition of IL-8 or blocking of IL-8 signaling by antibodies against either IL-8 (αIL-8) or its receptor CXCR1 (αCXCR1) in combination with TRAIL-influenced cell viability.

Cell viability analysis showed that IL-8 treatment neutralized the apoptosis-inducing power of TRAIL in L3.6pl cells and even more in L3.6plTR cells (Figure 5B, left panel). Conversely, blocking of CXCR1 or IL-8 in combination with TRAIL showed encouraging synergetic apoptosis-inducting effects compared to TRAIL alone, especially in L3.6plTR cells (Figure 5B, left panel). We also tested the effect of the combinations in sphere-forming assays and found that L3.6plTR cells were particularly sensitive to blocking of IL-8 signaling in combination with TRAIL (Figure 5B, right panel). Quantitative RT-PCR revealed that IL-8 autostimulated *IL-8* and *CXCR1* expression and contributed to TRAIL resistance by upregulation of the decoy receptor *DcR1* (Figure 5C). JNKi downregulated *c-JUN* and *DcR1* as expected and, surprisingly, lowered *CXCR1* levels considerably (Figure 5C), suggesting cross-links on multiple levels of these pathways. We confirmed by ELISA that TRAIL upregulates IL-8 secretion through JNK signaling and that IL-8 secretion can be blocked by JNKi (Figure 5D).

Together, our findings suggest the following model (graphically summarized in Figure 5E): Pancreatic CSCs escape TRAIL-induced apoptosis through activation of JNK signaling, which, in turn leads to increased IL-8 secretion and TRAIL resistance through downregulation of DR4/DR5 and upregulation of DcR1. IL-8 induces the expression of its cell surface receptor CXCR1, resulting in an autocrine feedback loop. This could presumably maintain TRAIL-resistance in pancreatic CSCs. On the other hand, these loops could be disrupted at multiple levels by JNKi (SP600125), αIL-8, or αCXCR1, thus restoring the TRAIL-sensitive pancreatic cancer subtype.

**Figure 6:**
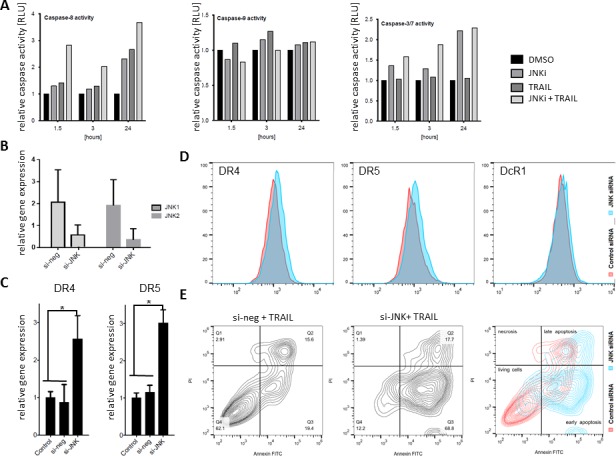
siRNA JNK interference sensitized L3.6pl to apoptosis **A.** Luminometer readings of purified recombinant caspase-8 enzyme were assayed in L3.6pl cells after 24-hour treatment with JNKi, TRAIL, or JNKi/TRAIL by Apoptosis-Caspase-Glo 8, 9 and 3/7 Assay. Each point represents the average of three wells. **B.** qRT-PCR of PDAC cells after siRNA to JNK shows significantly reduced *JNK1* and *JNK2* expression upon treatment. **C.** Gene expression of *DR4* and *DR5* after treatment with JNK siRNA for 48 hours. Shown are the relative values compared to control siRNA. *β-Actin* served as a housekeeping gene. **D.** Flowcytometry on JNK siRNA-treated PDAC cells (blue) on DR4 (9.3%), DR5 (12.6%) and DcR1 (0.1%) plotted on control siRNA (red) DR4 (5%), DR5 (8.3%) and DcR1 (0%) of labeled living cell population. **E.** L3.6pl transfected by control siRNA (left panel) and JNK siRNA (right panel), were treated with TRAIL (10ng/ml) for 48h. Cell death (upper, left), living cells (lower, left), and apoptosis (early lower right; late, upper right) were evaluated by flowcytometry with Annexin V-FITC/PI-staining. Combined plots on right panel shows JNK siRNA (blue) and control siRNA (red). Experiments were performed in triplicate. *p < 0.01.

Finally, we found that the extrinsic apoptosis pathway as measured by caspase-8 activity (TRAIL related extrinsic pathway) was clearly upregulated upon JNKi and TRAIL (Figure 6A left), whereas no significant changes in caspase-9 activity (intrinsic apoptosis pathway) could be detected (Figure 6A middle). Noticeable activity of the executioner caspases 3/7, especially after 24h and joint JNKi plus TRAIL treatment, was observed (Figure 6A, right).

### Specific silencing of JNK by siRNA predisposes PDAC cells to TRAIL-induced apoptosis by modification of death receptor expression

To furthermore scrutinize the effect of JNKi on pancreatic cancer cell lines, we selectively silenced SAPK/JNK gene expression by treatment with small interfering RNA (siRNA). JNK 1 and 2 interference resulted in lower JNK1 and JNK2 gene expression as demonstrated by qRT-PCR (Figure 6B). Moreover, we found that gene silencing with JNK siRNA led to a significant increase of DR4 and DR5 gene expression compared to control cells (Figure 6C). Following our hypothesis, we explored the death receptors surface protein expression upon siRNA to JNK by flowcytometry and noticed a significantly increased expression of DR4 (Absolute 4.3%, Relative 86.7%) and DR5 (Absolute 4.3%, Relative 51.2%) without a significant difference in DcR1 (Figure 6D). In accordance with previous results, siRNA to JNK plus low-dose TRAIL (10ng/ml) in L3.6pl cell line revealed a significant increase in early apoptosis compared to control siRNA plus TRAIL (68.8% vs. 19.4%) and a reduced living cells (12.2% vs. 62.1%) suggesting that effects detected on *small molecule inhibitor* use is specific (Figure 6E).

### Combination of JNKi and TRAIL reduces tumor growth *in vivo*

**Figure 7:**
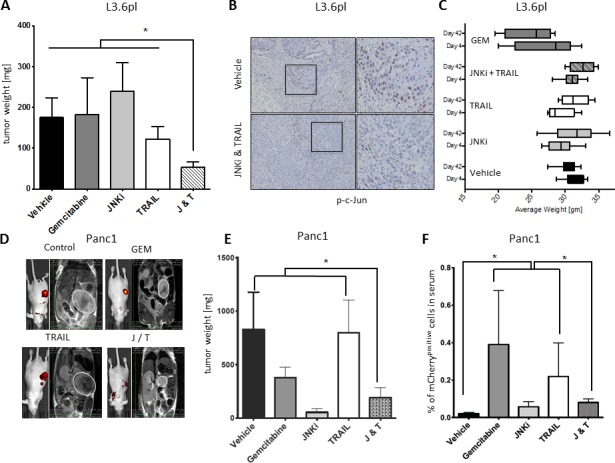
JNKi/TRAIL reduces tumor growth *in vivo* **A.** Tumor weight in mice inoculated with L3.6pl cells (*n* ≥ 6); treatments were started 14 days after tumor cell inoculation. **B.** Immunohistochemistry of phospho-c-Jun (brown nuclear staining) representative of activated JNK signaling on sections of L3.6pl tumors of **A.**. Magnification for better visibility is indicated by black squares. **C.** Weight of mice inoculated with L3.6pl before first treatment (day 4) and before end of study (day 42). **D.** *In vitro* imaging system (IVIS) images (left picture of each group indicates presence of tumor by yellow and red intensity) and MRI images (right picture of each group) of Panc1 tumors (for better visibility, PDAC tumors are encircled in white) of **E.** 7 days before mice were sacrificed. White bars on MRI indicate tumor length and width. **E.** Tumor weight of mice inoculated with Panc1 cells at the end of the study (treatments were started after tumor manifestation was confirmed by MRI; *n* = 5-7). **F.** mCherry-labelled Panc1 CTCs were analyzed by flow cytometry in RBC-depleted blood. Shown is relative number of CTCs in relation to the number of blood cells.

To mimic the clinical situation in which physicians encounter diverse pancreatic tumors in a number of patients, we carried out orthotopic xenotransplants with different PDAC cell lines. To test the efficacy of the combination of JNKi and TRAIL *in vivo*, we injected L3.6pl or MiaPaCa2 pancreatic cancer cells orthotopically into the pancreas of age-matched male, athymic nu/nu mice and initiated treatment 2 weeks after tumor cell inoculation. JNKi (1 mg/kg) was administered orally five times a week, TRAIL (1 mg/kg) intraperitoneally twice a week, and gemcitabine (80 mg/kg) twice a week (to permit comparison of JNKi and TRAIL treatments to standard therapy). After 4 weeks of treatment, we found that the weights of L3.6pl tumors in mice treated with the combination of JNKi and TRAIL (JNKi/TRAIL) were significantly lower than those in mice treated with vehicle or TRAIL alone (Figure 7A). MiaPaCa2 tumors showed a less prominent reaction to JNKi/TRAIL treatment in terms of tumor count and tumor weight (Supplementary Figure S4). However, in mice inoculated with MiaPaCa2 cells, the total number of metastases, a hallmark of CSCs, was significantly reduced in JNKi/TRAIL-treated mice compared to control mice as well (Supplementary Figure S4B). L3.6pl showed no evident metastasis. Interestingly however, the weight of animals tended to decrease upon Gemcitabine treatment, whereas JNKi, TRAIL or the combination thereof did not influence the weight of the mice, suggesting minimal therapeutic side effect *in vivo* (Figure 7C). As we observed *in vitro* (Supplementary Figure S1C), we found that p-c-Jun expression was profoundly reduced in sections of tumors treated with JNKi/TRAIL compared to vehicle control (Figure 7B).

To further investigate these findings, we treated firmly established tumors. Panc1 cells were orthotopically injected into age-matched male, athymic nu/nu mice, tumor growth was confirmed by magnetic resonance imaging (MRI) and IVIS (Figure 7D), and treatment was started after 28 days. We found markedly decreased tumor weights in mice treated with JNKi/TRAIL compared to mice treated with TRAIL alone or vehicle control (Figure 7E). CTCs in Panc1, that had been labeled with a lentiviral reporter (7xTcf-eGFP/SV40-mCherry (7TGC)) prior to orthotopic injection as reported before, were analyzed by flowcytometry in RBC-depleted whole blood showing a noticeable trend for chemo agents (Gemcitibine and TRAIL) to drive tumor cells into the circulation when compared to vehicle control (Figure 7F) [25, 30]. This effect was significantly reverted by combining JNKi to TRAIL treatment or by JNKi alone suggesting potent inhibitory potential of JNKi on the early metastatic steps of CSCs (Figure 7F). In fact, established tumors seemed to respond early to treatment with JNKi/TRAIL as determined by *in vitro* imaging system and MRI (Figure 7D).

Together, these findings convincingly demonstrate that low-dose JNKi/TRAIL treatment significantly reduces tumor growth both in TRAIL-sensitive tumors and even reduce tumor size, release of circulating tumor cells, and incidence of metastasis in TRAIL-resistant tumors to a greater extent (Supplementary Table S1).

## DISCUSSION

Antineoplastic strategies against both bulk tumor cells as well as against tumor stem cells are imperative for successfully reducing tumor size and improving overall patient survival. This is especially crucial in cancers that are detected late in the course of the disease and in tumors that exhibit a relative drug resistance with a high propensity for metastasis. In PDAC, one further key to successful treatment is a better understanding of the heterogeneity of the tumor and its drivers.

In the current study, we examined the role of the JNK pathway in PDAC - a pathway that is activated by inflammatory or hypoxic stimuli and is involved in apoptosis regulation [22].

Previous reports have suggested that JNK signaling regulates cancer stemness and presents an escape pathway from apoptosis, with the majority of these data derived from studies in hepatocellular carcinoma [31, 32]. Moreover, it has been shown that stem-like glioma cells depend on JNK signaling, which suggests this pathway as an attractive target for therapeutic strategies [8, 33]. Interestingly, recent studies indicate that oncogenic KRAS forms a critical axis with the JNK pathway that can regulate pancreatic tumor formation [9].

In this work, we found that low-dose JNK inhibition (JNKi) significantly decreased growth patterns in different pancreatic cancer cell lines in adherent culture (Figure 1A, 1B and Supplementary Figure S1A, B) or nonadherent, CSC-enriched sphere culture (Figure 2B-2C and Supplementary S2B). This suggests that JNK and its downstream targets are important in pancreatic cancer for proliferative activities of differentiated bulk tumor cells as well as for regulation of self-renewal in pancreatic CSCs (Figure 1C and Supplementary Figure S1C, D). Moreover, we found that JNKi not only reduced JNK target gene expression (Figure 1D and Supplementary Figure S2A) but also significantly inhibited CSC markers in bulk tumor cells (Figure 1D and Supplementary Figure S2A) and in CSC-enriched spheres (Figure 2D). The latter observation explains JNKi's potent effect on reducing the self-renewal capacity in CSCs.

In an attempt to potentiate JNKi's antiproliferative effect for a translational antitumor approach, we combined low-dose JNKi with a natural apoptosis-inducing agent. Here, we chose TRAIL, which is produced by many tissues and mainly induces extrinsic apoptosis in neoplastic cells because of their expression of the functional TRAIL receptors DR4 and DR5 [34]. In the case of PDAC, TRAIL-induced cell death is primarily mediated by DR4 [26]. However, many tumors also develop resistance mechanisms by upregulating intrinsic inhibitors of apoptosis, e.g. c-FLIP or the nonfunctional Decoy-TRAIL receptors DcR1 or DcR2 [35, 36]. In hepatocellular carcinoma, it has been reported that JNKi restored sensitivity to the apoptosis-inducing ligand to CD95, however only in considerably higher dosages than used in the present study [23].

Our results demonstrate that the combination of low-dose JNKi and TRAIL drastically reduces cell viability in adherent, bulk tumor cells (Figure 3B) and, to an even larger degree, in CSC-enriched spheres (Figure 3C), which are intrinsically more resistant to TRAIL (Figure 3A). Of note, low-dose JNKi is even able to overcome acquired TRAIL resistance in PDAC and its spheres (Figure 4B, 4D) by upregulating the expression of functional TRAIL receptors DR4 and DR5 (Figures 3D, 6C, 6D and Supplementary Figure S2D) and downregulating the decoy receptors DcR1 and 2. Low-dose JNK plus TRAIL in pancreatic cancer cell lines revealed a significant increase in apoptosis compared to control by extrinsic caspase-8 activity (Figures 3C, 6A, 6E and Supplementary S3D).

To further test the suitability of the JNKi-TRAIL combination for possible future clinical use, we treated several orthotopic pancreatic tumors with varying TRAIL susceptibility with JNKi, TRAIL, or a combination thereof. In an animal model of orthotopic xenografts, tumors were treated successfully with very low doses of JNKi/TRAIL combination therapy, showing >70% reduction in tumor weight compared to JNK (11%) or TRAIL (10%) alone, suggesting a synergistic rather than an additive effect (Figure 7 and Supplementary Table S1). Only in one cell line (MiaPaCa2), were low doses of both JNKi and TRAIL not able to significantly impact *in vivo* tumor growth, possibly due to treatment low concentration. Since we used low doses of both JNKi and TRAIL in our orthotophic pancreatic model - up to 40 fold lower than prior glioma model [37] [27] - further studies should evaluate if an increase in dosage or duration of treatment would be beneficial in highly resistant tumors, especially in patient-derived xenograft model systems.

Importantly, we found a reduction in circulating tumor cells and metastatic spread, indicative of a significant anti-metastatic-stem-cell effect for this combination *in vivo* (Figure 7D and Supplementary Table S1). Furthermore, we demonstrate that JNKi, TRAIL, and the combination of these two agents (in doses up to five times of those used in our *in vivo* treatment experiments) had no effect on proliferation, survival, and, most importantly, the functional differentiation capacity of normal tissue-resident stem cells (Supplementary Figure S3A-C). This indicates that the concept of JNKi/TRAIL combination treatment could be clinically well tolerated by pancreatic cancer patients. Moreover, even under hypoxic conditions, which are typically found in poorly vascularized cancers such as PDAC and which also activate stress pathways, regular adult stem cells remained unaffected by JNKi and TRAIL. In contrast, PDAC tumor cell lines showed significant levels of cell death, including in TRAIL-resistant tumor cell lines at these low-dose combinations (Supplementary Figure S3D).

Recent biomarker profiling of pancreatic cancer suggests that functional p38 MAPK activity inhibits JNK and thus improves overall survival, thus corroborating our approach [38]. However, this report did not characterize the missing link between the different pathways. Here, we identified IL-8 as the critical link between the JNK pathway, TRAIL resistance, and cancer stemness in PDAC. It was previously shown that IL-8 and its receptor CXCR1 are protagonists especially in breast cancer stem cells [39, 40]. Moreover, in a prostate cancer model, Wilson et al. showed that endogenous IL-8 or drug-induced heightened secretion of IL-8 substantially reduced drug sensibility [29] and, in a similar manner, IL-8 treatment was shown to induce relative TRAIL-resistance in the ovarian cancer cell line OVCAR3 [41]. In our study, we show that TRAIL-induced IL-8 secretion improved cell survival by increasing the expression of TRAIL-decoy receptors DcR1 and 2 and reducing death receptors DR4 and 5 when facing TRAIL; the latter effects were reversible by JNKi (Figure 5B). In turn, blocking of IL-8 signaling by antibodies against IL-8 or its receptor CXCR1 reduced survival and cancer stemness significantly (Figure 5B). JNKi interfered with this axis to some extent by decreasing CXCR1 expression (Figure 5C).

In summary, our findings show that the JNK pathway is an important regulatory pathway in pancreatic cancer stem cells. Its inhibition offers a novel, selective approach to treat pancreatic cancer by targeting parental pancreatic cancer cells and, to an even higher degree, affecting the growth and physiology of pancreatic cancer stem cells. Most importantly, we provide evidence from our experiments that this combined sensitizing treatment has a considerable safety window, as the physiology of normal tissue-resident stem cell is not impacted, even at much higher drug doses as used in the animal study.

In our study, we administered TRAIL systemically by intraperitoneal injections. However, it is known that a further increase in systemic levels of TRAIL can be associated with side effects. Ours and others previous studies have shown that stem cells home to tumor sites [13, 42] described as a “never healing wound” [43] after i.v. application. Hence, genetically modified mesenchymal stem cells that overexpress TRAIL could selectively increase local TRAIL levels in the tumor environment [44, 45]. This might have potential merits, especially in combination with systemically applied low-dose JNK inhibition. The approach we describe is representative of the next generation of cancer therapy as it aims to be a more selective, targeted, efficacious and possibly safer mode of treatment.

## MATERIALS AND METHODS

### Cell isolation and culture

Panc1 (obtained from American Type Culture Collection), MiaPaCa2, L3.6pl, Patx1, and HS766T pancreatic cancer cells (kind gifts of Dr. Kenji Yokoi) were maintained in minimum essential medium (MEM; Corning Incorporated) supplemented with 10% fetal bovine serum (FBS; Atlanta Biologicals), 1% penicillin-streptomycin, L-glutamine, MEM nonessential amino acids (all from Corning), and MEM vitamin solution (Gibco) at 37°C in 5% CO_2_. Medium was changed every 3 days, and cells were passaged before reaching 80% confluence. For experiments under hypoxic conditions, cells were cultured in the humidified modular hypoxia chamber (Billups-Rothenberg), which contained a 95% N_2_ and 5% CO_2_ mixture.

### Sphere culture and sphere-forming assay

Sphere-forming medium consisted of MEMα supplemented with L-glutamine, putrescine, insulin (all from Sigma-Aldrich), epithelial growth factor (20 ng/mL), basic fibroblast growth factor (10 ng/mL), and B-27 supplement (Gibco). For first generation, attached cells were trypsinized, washed twice with PBS, and seeded in sphere-forming medium as single cell suspensions with clonal density (5,000-10,000 cells/mL) on ultra-low-attachment plates (Corning). After 7 to 10 days, spheres were harvested by gravitation in a tube, trypsinized, washed twice with PBS, and reseeded as described above for the next higher generation.

To quantify sphere-forming ability, cells were prepared as described in the preceding paragraph and seeded in 96-well ultra-low-attachment plates at 500 to 1000 cells per well. Medium was supplemented with 1% methylcellulose to prevent cell-cell attachments. Medium was added or renewed every 3 days, and spheres were quantified at day 10 to 12.

### Isolation of human adipose-tissue-derived stem cells

Human subcutaneous adipose tissue was obtained from patients undergoing elective lipoaspiration with informed consent (The University of Texas MD Anderson Cancer Center Institutional Review Board registrations IRB00001035, IRB00003657, IRB00004920, and IRB00006075). Adipose tissue was washed thoroughly, minced, and incubated with Ringers lactate containing a combination of collagenase I and II and a neutral protease (Matrase ^TM^ Reagent, InGeneron Inc. Houston TX) in a Tissue Processing Unit (Transpose RT^TM^ System, InGeneron Inc. Houston TX) for 30 minutes at 40°C. Subsequently, the cell suspension was filtered through a 100-μm filter, washed twice, and then centrifuged at 600 rpm for 5 minutes. The adipose stromal vascular fraction was resuspended in αMEM with 20% FBS, L-glutamine, and penicillin-streptomycin-amphotericin B (Sigma-Aldrich) at 37°C in 5% CO_2_. Red blood cells in the supernatant and nonadherent cells were removed after 48 hours. For all experiments shown, human subcutaneous adipose tissue-derived cells were used prior to passage 6.

### Materials

Jun N-terminal kinase inhibitor II SP600125 (JNKi) was obtained from Calbiochem, JNK-IN-8 from Selleckchem, recombinant human TRAIL (rhTRAIL) from R&D Systems, and Gemcitabine from Elly Lilly. Products were reconstituted as recommended by the manufacturer.

The following antibodies were used: phospho-c-Jun (Cell Signaling Technologies), CD133-APC (Miltenyi Biotech), SSEA1-FITC (Santa Cruz Biotechnology, Inc.), DR4 antibody-PE Mouse IgG1B, DR5 antibody-FITC Mouse IgG2B and DcR1 antibody-APC Mouse IgG1 (R&D Systems).

Small interfering RNA for SAPK/JNK (#6232) and control (#6568) was obtained from SignalSilence ^®^ (Cell Signaling Technologies).

### MTT assay

Cells were seeded in a 96-well plate at a density of 4000 to 5000 cells per well (70%-80% confluence) in triplicate. Non-adherent cells were washed with PBS, and different substrate dilutions were added. After 24 hours, 3-(4,5-dimethylthiazol-2-yl)-2,5-diphenyltetrazolium bromide (MTT) assay (Roche) was performed according to the manufacturer's instructions. Results were measured at 570 nm and background at 650 nm on a microplate reader (Molecular Devices).

### Invasion assay

Pancreatic cancer cells were pretreated in six wells for 24 hours with JNKi or control medium. Viable cells were then transferred to 24-well matrigel-coated invasion chambers with 8-mm pore size (BD Biosciences) in MEM with 1% FBS in the upper chamber and 500 μL of medium with 10% FBS in the lower chamber as a chemoattractant. After 24 hours, medium was removed, inserts were washed with PBS, and noninvaded cells were carefully removed. Cells on the bottom of the insert were fixed with ice-cold MetOH for 10 minutes, stained with crystal violet, and counted by light microscopy.

### Colony-forming assay

Cells were pretreated with JNKi as described in the preceding section. Five hundred viable cells were seeded in triplicate into a six-well plate with 3 mL of medium and incubated without change of medium. After 10 days, colonies were washed, fixed, and stained with crystal violet. Colonies were counted in four different view fields.

### Two-dimensional tumor cell migration scratch assay

Ninety-percent-confluent pancreatic cancer cell layers were scratched with the tip of a 10-μL pipette, washed with PBS, and further cultured with and without JNKi. Gap distances were measured by light microscopy at 0 hours, 16 hours, and 32 hours. Migration movement was measured in nine different fields. For immunofluorescent staining, the scratch assay was performed on a glass cover slide, and cells were then fixed, permeabilized with 80% EtOH, and stained with p-c-Jun primary antibody (Cell Signaling), Alexa-594-conjugated anti-rabbit secondary antibody, and DAPI for counterstaining of nuclei.

### Acridine orange/ethidium bromide staining

According to a protocol adapted from Todaro et al. [10], attached cells or spheres were washed with PBS, stained with acridine orange/ethidium bromide, and visualized immediately with fluorescent microscopy.

### Flowcytometric and fluorescence microscopy evaluation of apoptosis

For the distinction of early and late apoptotic or necrotic events after treatment as described in the text, flowcytometry with the FITC Annexin V Apoptosis Detection Kit II (BD Pharmingen^TM^) was carried out. Briefly, after treatment, cells were trypsinized, stained for 15 min at RT (25°C) in the dark and analyzed within 1hr according to the manufacturer's instructions. Flowcytometry protocol for death receptor antibodies required the following modifications, briefly, 24 hours after incubation in different conditions, cells was washed in PBS without Ca^2+^ and Mg^2+^ and harvested using Sodium Citrate solution. Cells were blocked 15 min at 4°C in 0.01% BSA and incubated at 4°C with the different antibodies. Cell were then washed twice with PBS and maintained on ice until flow analysis within the hour.

### Quantitative reverse transcription-PCR

For total RNA extraction, cells were homogenized with TRIzol (Invitrogen). Phase separation was performed by the addition of chloroform and subsequent centrifugation steps. Aqueous phase of samples was collected, and RNA was precipitated by isopropyl alcohol. After washing, RNA was redissolved in DEPC-treated water, and RNA quality and quantity were measured with a Nanodrop ND-1000 Spectrophotometer (Thermo Scientific). For cDNA synthesis, the iScript Reverse Transcription Supermix (Bio-Rad) was used according to the manufacturer's protocol, and the reaction mix was incubated in a thermal cycler (Bio-Rad MyIQ Single-Color RT-PCR Detection System iCycler) with the following protocol: priming (5 minutes at 25°C), reverse transcription (30 minutes at 42°C), and reverse transcription inactivation (5 minutes at 85°C). qRT-PCR was performed using iQ SYBR Green Supermix (Bio-Rad) according to the following protocol: initial denaturation and enzyme activation (1 cycle at 95°C for 3 minutes), denaturing (40 cycles at 95°C for 15 seconds) with annealing and extension (40 cycles at 55°C for 30 seconds), and melting curve (1 cycle at 55°C-95°C in 5-°C increments for 30 seconds). The C_t_ (cycle threshold) value was measured in absolute quantification (of cycles of amplification) and compared to *β-actin,* which served as a housekeeping gene. Primer sequences of primers used in this study can be seen in Supplementary Table S2.

### PathScan p-SAPK/JNK sandwich ELISA

Protein lysates were obtained from adherent cells or spheres after 1-hour of incubation with JNKi and/or TRAIL. Protein quantification was performed, and PathScan Sandwich ELISA Antibody Pair (Cell Signaling Technologies) was performed according to the manufacturer's instructions.

### Human CXCL8/IL-8 immunoassay

Cell culture supernatant was obtained after 24-hour treatments in triplicate. A human CXCL8/IL-8 Quantikine ELISA kit (R&D Systems) was used to measure human IL-8 according to the manufacturer's protocol. An IL-8 standard curve was performed to determine concentrations (in pg of cytokine per μL).

### Caspase activity assay

After 24 hours, Caspase-Glo^®^ (8, 9 and 3/7) assay protocol (Promega) was performed according to the manufacturer's instructions in a 96-well plate at a density of 4000 to 5000 cells seeded per well (70%-80% confluence) in triplicate on different substrate dilutions that had been previously added. Results were measured by adding luciferase substrate which generates a “glow-type” luminescent signal. MG-132 inhibitor was used to eliminate none-specific background. Luminometric read out was performed after 30 minutes.

### siRNA transfection assay

Cells were transfected with 100nM SAPK/JNK siRNAI (#6232) or control siRNA (#6568), from SignalSilence Cell Signaling Technologies using FuGENE^®^ Transfection Reagent (Promega), following manufacturers recommendations. For the flowcytometric and RT-qPCR experiments, cells were harvested 48 h hours after transfection and followed prior mentioned methods.

### Differentiation assay

Adipose tissue-derived stem cells were seeded in the following concentrations: adipogenic differentiation, 1×10^4^ cells/cm^2^; chondrogenic differentiation, 1.6×10^7^ cells/cm^2^; and osteogenic differentiation, 5×10^3^ cells/cm^2^. After a 2-hour incubation with 20% FBS-containing medium, cells were washed, and the respective differentiation media were added (Invitrogen StemPro differentiation kits). Differentiation media were changed twice a week. After 14 to 21 days, cells were fixed with 4% formaldehyde for 30 minutes and stained with Oil Red O for lipid vesicles (adipogenic differentiation), Alcian Blue for proteoglycans (chondrogenic differentiation), and Alizarin Red S for calcium deposits (osteogenic differentiation) as reported previously [46].

### Animal studies

Age-matched male swiss nu/nu mice (6-8 weeks old) were injected orthotopically with pancreatic tumor cells. All procedures were performed in accordance with the guidelines of the Institutional Animal Care and Use Committee at The University of Texas MD Anderson Cancer Center (ACUF Protocol # 12-12-12631). Animals were anaesthetized with isoflurane anaesthesia (1%-3% via inhalation), and an incision was made in the left abdominal flank. The spleen was located and extracted, and 1×10^6^ pancreatic cells in 50 μL of PBS were injected into the underlying tail of the pancreas. The abdominal wall was closed with sterile absorbable sutures, and wound clips were applied to the skin. Animals were monitored daily and after two weeks of untreated tumor growth randomly assigned to different treatment groups: control (no treatment), JNKi (1 mg/kg) was administered by oral gavage five times per week; gemcitabine (80 mg/kg) or TRAIL (1 mg/kg) was injected intraperitoneally with a 27-G needle two times per week or JNKi and TRAIL together at the dosages indicated above. Weight, tumor growth, and health status were clinically followed for 4 weeks. At day 42, animals were euthanized, and blood and tissues were collected for postmortem analysis.

### Statistical analyses

Results are expressed as the mean ± standard of the mean. All statistical comparisons were made with a standard t-test or t-test with Welch's correction (where indicated), using biostatistics software from GraphPad Prism. For all comparisons, *p* < 0.05 was considered statistically significant.

## SUPPLEMENTARY MATERIAL FIGURES AND TABLES


