# Supplementary material for: JNK pathway inhibition selectively primes pancreatic cancer stem cells to TRAIL-induced apoptosis without affecting the physiology of normal tissue resident stem cells
Source: Oncotarget. 2016 Jan 28;7(9):9890–906. doi: 10.18632/oncotarget.7066 (PMC4891091; doi:10.18632/oncotarget.7066)
Supplement: Supplementary file 1 [file oncotarget-07-09890-s001.pdf]

JNK pathway inhibition selectively primes pancreatic cancer stem cells to TRAIL-induced apoptosis without affecting the physiology of normal tissue resident stem cells

Supplementary Material

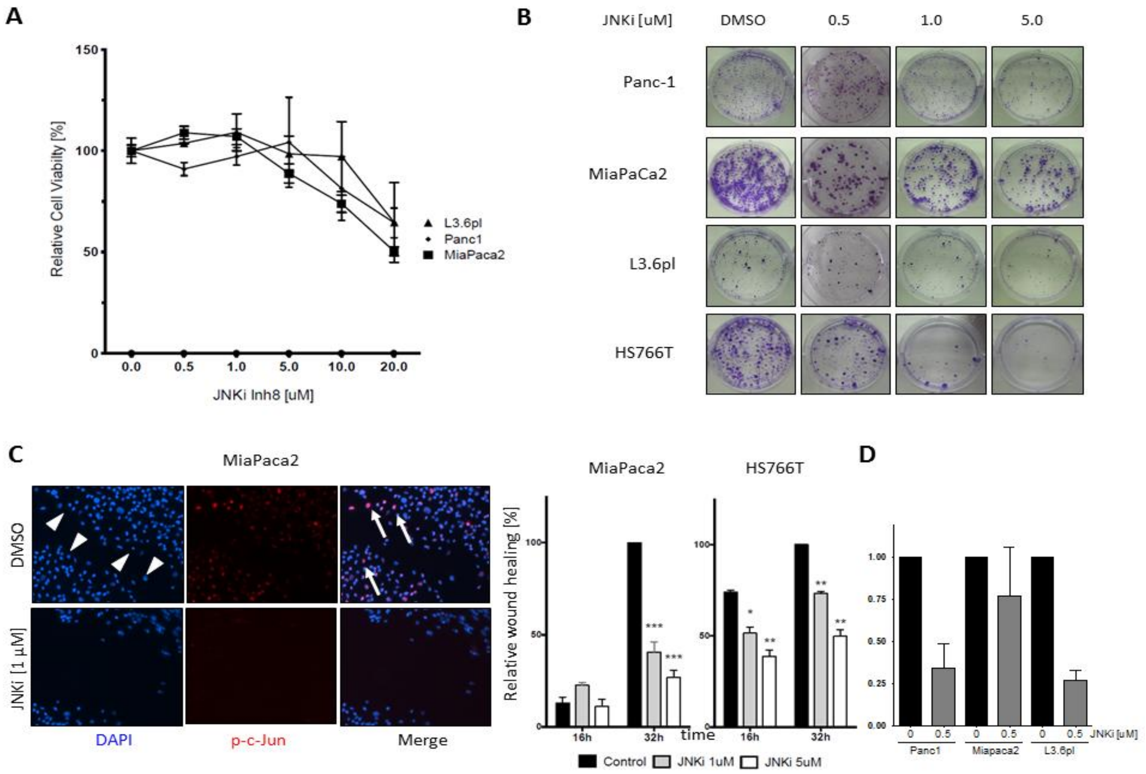

Figure S1: (A) Cell viability of Panc1, MiaPaCa2, and L3.6pl pancreatic cancer cells was determined by MTT assay after treatment with increasing doses of JNKinh8 for 24 hours. Experiments were performed in triplicate. Figure related to Fig. 1A. (B) Brightfield micrographs of colony formation after 10-day incubation of cells with JNKi in six-well plates and staining of colonies with crystal violet. Figure is related to Fig. 1B. (C) Immunofluorescent staining of wound-healing assay for phospho-c-Jun (p-c-Jun; red). Cells were counterstained with DAPI (blue). White arrowheads indicate the borders of the wound (upper left); white arrows show cells with p-c-Jun expression in the

immediate vicinity of the wound (upper right). Four different fields were analyzed per time point and treatment. Shown are the mean lengths of the gap closure in relation to the length of the original closure determined at 0 hours. Figure related to Fig. 1C. (D) Relative invasion of Panc1, MiaPaCa2, and L3.6pl cells. Invading cells were counted in four different view fields and presented as the mean ± SD.

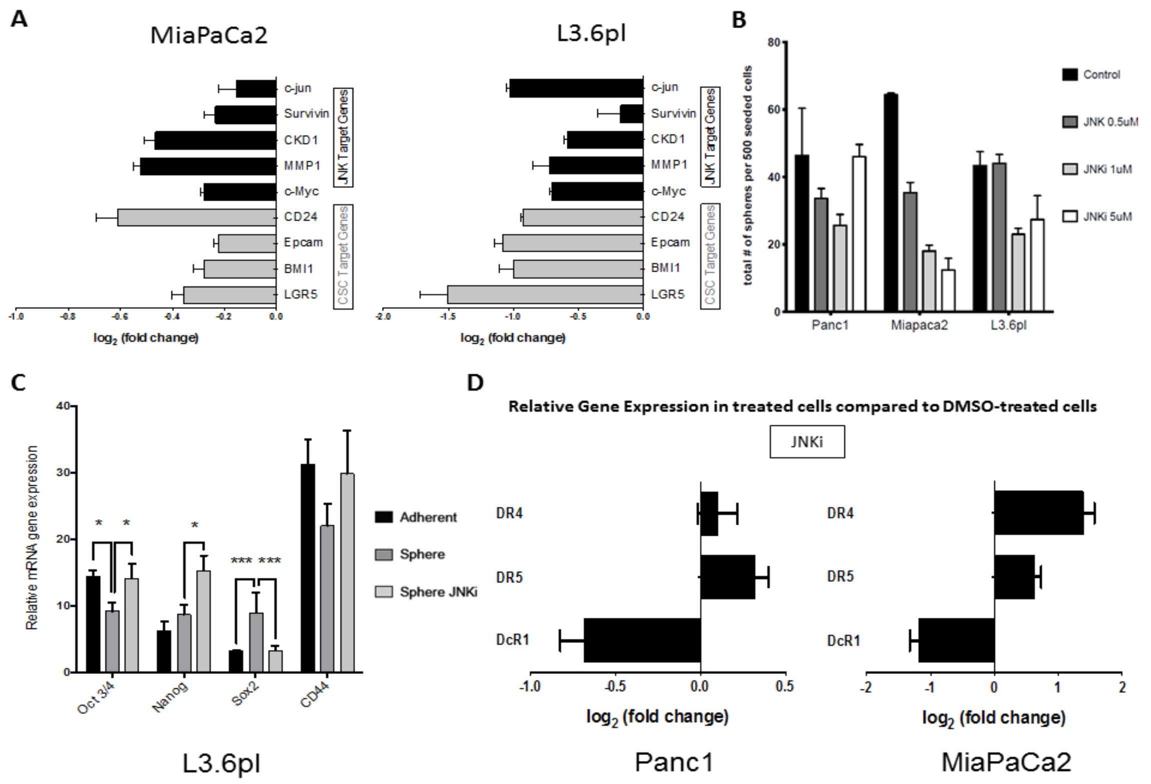

Figure S2: (A) Expression of JNK target genes (black bars) or CSC target genes (grey bars) in JNKi-treated cells (0.5 µM) relative to expression in untreated cells as determined by qRT-PCR. Values of genes were standardized to the respective values of housekeeping genes. Related to Fig. 1D. (B) Sphere-forming ability after treatment with JNKi at one time dose. Spheres were counted after 10 to 12 days. (C) qRT-PCR of CSC markers in parental L3.6pl cells, spheres, and spheres treated with JNKi (0.5 µM). Figure related to Fig. 2D. (D) Gene expression of *DR4, DR5,* and *DcR1* in Panc1 and MiaPaCa2 cells after treatment with JNKi for 24 hours. Shown are the relative values compared to untreated controls. Figure related to Fig. 3D. Experiment was performed in triplicate. $^{**}p < 0.01$. $^{***}p < 0.001$.

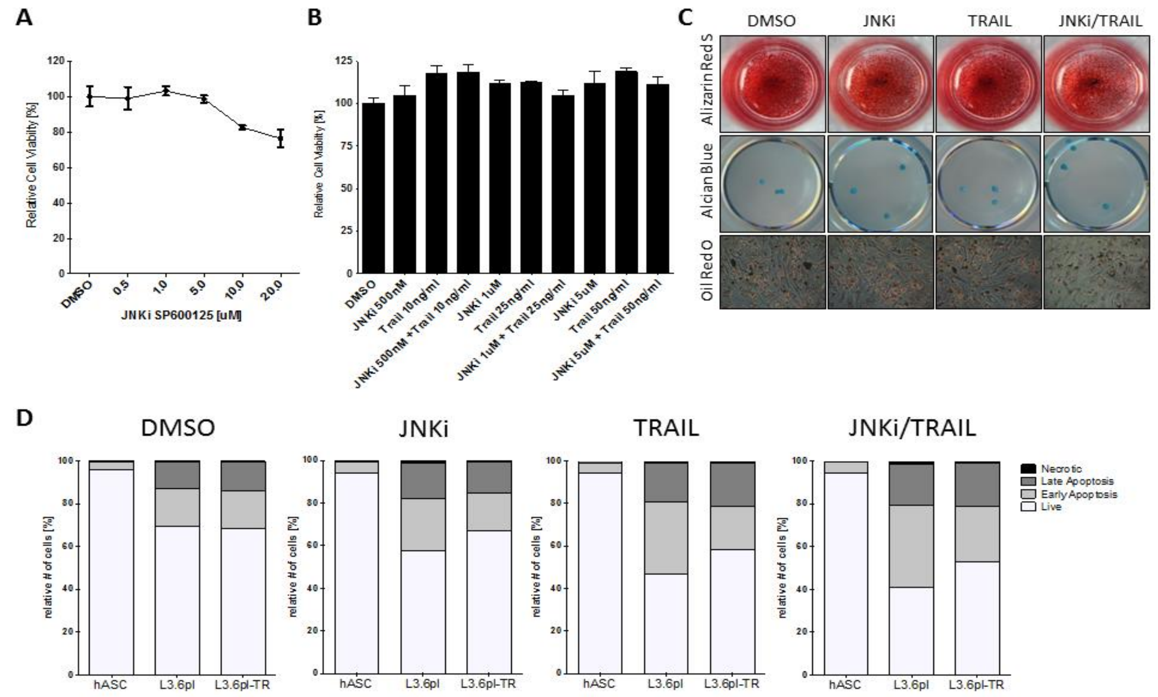

Figure S3: (A) MTT proliferation assay of adipose tissue-derived stem cells (ASCs) with increasing doses of JNKi. Experiment was performed in triplicate. (B) Relative cell survival of ASCs after exposure to JNKi and/or TRAIL with doses up to five times those used in pancreatic cancer treatment. Experiment was performed in triplicate. (C) Differentiation assays of ASCs into adipocytes, chondrocytes or osteoblasts. Confirmation of terminal differentiation was carried out by standard staining procedures (Alizarin Red, Alcian Blue, and Oil Red O). (D) hASC, L3.6pl or L3.6pl-TR were cultured under hypoxic conditions (95% N_2 and 5% CO_2 mixture) and DMSO, JNKi, TRAIL, or JNKi/TRAIL for 48h. Cell death (necrosis, black) and apoptosis (early, light grey; late, dark grey) were evaluated by Annexin V-FITC/PI-staining.

A

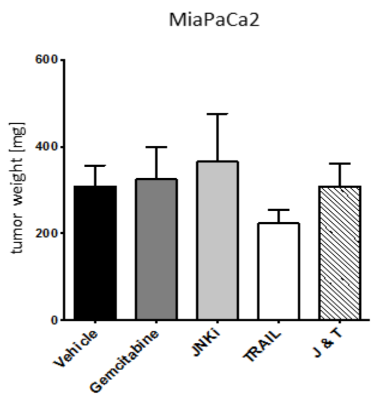

B

MiaPaCa2 Group	# of tumors at 6 weeks	6-weeks tumor presence %	liver metastasis	spleen met	peritoneal met	kidney	Total mets # / [%]
Vehicle	10 / 10	100	1	5	3	1	10 / 100 %
GEM	5 / 5	100	1	3	1	0	5 / 100 %
JNKi	4 / 4	100	1	1	0	0	2 / 50 %
TRAIL	10 / 10	100	2	0	1	0	3 / 30 %
JNKi / TRAIL	9 / 10	90	0	5	0	0	5 / 50 %

Figure S4: (A) MiaPaCa2 cells were orthotopically injected and treatment was started after 2 weeks similar to Fig. 7A (n = 10 in vehicle, TRAIL, and JNKi/TRAIL groups; n = 5 in gemcitabine and JNKi groups). (B) Table of tumor take and metastastatic index in MiaPaCa2.

Table S1: Metastatic index in Panc1 orthotopic xenograft. # of tumors at 6 weeks means tumors encountered at point of sacrification of mice; 6-weeks tumor presence % is equivalent to tumors encountered divided by total number of mice per group; liver/spleen/peritoneal/kidney metastasis shows the number of mice with macroscopic metastasis in the respective organ.

Panc1 Group	# of tumors at 6 weeks	6-weeks tumor presence [%]	liver metastasis	spleen met	peritoneal met	kidney	Total mets # / [%]
Vehicle	9 / 10	90	1	5	1	0	7 / 70 %
GEM	5 / 5	100	0	0	0	0	0 / 0 %
JNKi	3 / 4	75	0	1	0	0	1 / 25 %
TRAIL	9 / 10	90	0	2	0	0	2 / 20 %
JNKi / TRAIL	5 / 10	50	1	1	0	0	2 / 20 %

Table S2: Primer list for PCR. Primer, Forward 5' to 3' and Reverse 5' to 3'.

Primer	Forward 5' to 3'	Reverse 5' to 3'
JNK1	TGTGTGGAATCAAGCACCTTC	AGGCGTCATCATAAAACTCGTTC
JNK2	GAAACTAAGCCGTCCTTTTCAGA	TCCAGCTCCATGTGAATAACCT
c-jun	ACAGCTTCATGCCTTTGTAA	CTCAGAGTGCTCCAAATCTC
Survivin	CGCCAGTGTTTCTTCTGCTT	CCGGACGAATGCTTTTTATG
CKD1	CCGCTGGCCATGAACTACCT	ACGAAGGTCTGCGCGTGTT
MMP1	AAAATTACACGCCAGATTTGCC	CAGAGTTGGAAGGCTTTCTCAAT
MYC (c-Myc)	TCAAGAGGCGAACACACAAC	GGCCTTTTCATTGTTTTCCA
CD24	AAACAACAACTGGAACTTCAAGTAACTC	GGTGGTGGCATTAGTTGGATTT
BMI1	GGAGACCAGCAAGTATTGTCCT	CATTGCTGCTGGGCATCGTAAG
LGR5	CACCTCCTACCTAGACCTCAGT	CGCAAGACGTAACTCCTCCAG
Oct3/4	CTTGCTGCAGAAGTGGGTGGAGGAA	CTGCAGTGTGGGTTTCGGGCA
NANOG	AGTCCCAAAGGCAAACAACCCACTTC	TGCTGGAGGCTGAGGTATTTCTGTCTC
SOX2	TGCGAGCGCTGCACAT	CCGGCGGAAAACCAAGACGCT
CD44	TGCCGCTTTGCAGGTGTAT	GGCCTCCGTCCGAGAGA
DR4	ACCTTCAAGTTTGTCGTCGTC	CCAAAGGGCTATGTTCCCATT
DR5	GCCCCACAACAAAAGAGGTC	AGGTCATTCCAGTGAGTGCTA
DcR1	TCCCCAAGACCCTAAAGTTCG	GGCAGAGTAAGCTAGGACTGG
IL-8	ACTGAGAGTGATTGAGAGTGGAC	AACCCTCTGCACCCAGTTTTC
CXCR1	CTGACCCAGAAGCGTCACTTG	CCAGGACCTCATAGCAAACTG
β-actin	ACCCTGAAGTACCCCATCG	CACCGGAGTCCATCACG
